# Suppression rather than activation of the integrated stress response (GCN2–ATF4) pathway extends lifespan in the fly

**DOI:** 10.1073/pnas.2518812123

**Published:** 2026-04-28

**Authors:** Miriam S. Götz, Dan J. Hayman, Gracie Adams, Fumiaki Obata, Mirre J. P. Simons

**Affiliations:** ^a^School of Biosciences, University of Sheffield, Sheffield S10 2TN, United Kingdom; ^b^RIKEN Center for Biosystems Dynamics Research, Kobe, Hyogo 650-0047, Japan; ^c^Laboratory of Molecular Cell Biology and Development, Graduate School of Biostudies, Kyoto University; Kyoto 606-8501, Japan

**Keywords:** integrated stress response, borrelidin, tRNA, transcriptomics, aging

## Abstract

Stress response pathways are emerging as conserved modulators of lifespan. The prevailing hypothesis is that activation of stress-responsive pathways, including the amino acid deprivation arm of the integrated stress response (ISR; the GCN2–ATF4 pathway), is prolongevity. Activation of *ATF4* orthologs extends lifespan in *Saccharomyces cerevisiae* and *Caenorhabditis elegans*, but its role in other longer-lived organisms remains unclear. We comprehensively tested the role of the GCN2–ATF4 pathway in longevity in the fly (*Drosophila melanogaster*) for the first time. We used conditional genetic manipulation of *dGCN2* and its downstream effector *Drosophila ATF4* (*crc*; *dATF4*). In contrast to previous studies, we show that overexpression of *dGCN2* and *dATF4* significantly reduces lifespan, while knockdown (in vivo RNAi) of *dATF4* extends lifespan. We confirmed that dATF4 activity was successfully modulated using a fluorescent *dATF4* activation reporter. Borrelidin, a tRNA synthetase inhibitor, significantly reduced lifespan in a both *dATF4* and diet-dependent manner, independent of microbial load, showing our modulation of *dATF4* altered nutrient to ISR signaling. We further conducted long-read RNA sequencing and found that our manipulation of *dATF4* changed global transcription in opposite directions, including known *ATF4* target genes. Enrichment analysis revealed that *dATF4* overexpression may drive metabolic stress, while *dATF4* knockdown may upregulate proteostasis and DNA repair pathways. Our work reveals that *ATF4* may exhibit a dual, dose-, and context-dependent role in aging. Chronic *dATF4* activation is detrimental in flies, while chronic suppression is prolongevity. The GCN2–ATF4 pathway thus qualifies as a modifiable control of lifespan with cross-species relevance.

## Significance

Recent progress in geroscience suggests that targeting broad aspects that underlie the biology of aging could prevent many age-related diseases simultaneously. One such treatment is the activation of stress response pathways. Recently, activation of the integrated stress response (ISR) orchestrated by the transcription factor ATF4 has been suggested to be prolongevity, but this has received limited testing beyond yeast and *Caenorhabditis elegans*. We tested *ATF4* in the fly and found that suppression rather than activation extends lifespan. We suggest that the exact level and duration of modulation of the ISR will require context-specific optimization. Our work further positions the GCN2–ATF4 pathway as a modifiable control of aging, with relevance across species.

Stress response pathways are emerging as conserved modulators of lifespan (1, 2). Mild exposure to stress leads to increased stress resistance and lifespan extension through hormesis (3). The activation of stress response pathways has further been suggested to be required for the longevity extension observed in many long-lived mutants (most clearly demonstrated in *Caenorhabditis elegans*; 4). Independently, overexpression of pathways that regulate stress responses (such as FOXOs, Nrf2, HIF; 5–9) and end-products of stress pathways such as heat-shock proteins (10), may be sufficient to extend lifespan (4). The pathway that may be responsible for these shared stress responses, the integrated stress response (ISR), a conserved stress-activated pathway, has been implicated in lifespan regulation across species (11, 12).

The ISR is activated by four kinases [General control nondepressible 2; GCN2, Protein Kinase R (PKR)-like endoplasmic reticulum kinase; PERK, PKR, and heme regulated inhibitor; HRI] in response to different types of stress (amino acid starvation, ER stress, viral infection, and heme deprivation, respectively; 13). Detection of stress [e.g., amino acid (AA) deficiencies via uncharged tRNAs and independently of tRNA via ribosomal arrest in the case of GCN2; 14, 15] by each of these kinases leads to phosphorylation of eukaryotic Initiation Factor 2 alpha (eIF2α; 16). Phosphorylation of eIF2α leads to a general reduction in global protein synthesis while at the same time selectively enhancing translation of Activating Transcription Factor 4 (ATF4) by enabling ribosomes to bypass inhibitory upstream Open Reading Frames (uORFs). However, mechanisms that activate ATF4 independent of eIF2α phosphorylation, although less well studied, can be important (17). Surprisingly, restriction of tyrosine (Tyr), a nonessential amino acid, activates ATF4 independently of GCN2, yet the effects of Tyr on lifespan are independent of ATF4 (18, 19). Upon activation ATF4 acts as a key regulator of cell fate (20) by driving a broad transcriptional response thought to help cells adapt to stress (21). Importantly, ATF4’s target genes can either promote autophagy or apoptosis, depending on the intensity, duration, and cellular context of the response (22).

Evidence from multiple species suggests that orthologs of *ATF4* can affect lifespan (Table 1). However, experimental evidence linking *ATF4* to lifespan is currently limited to its orthologs *GCN4* in *Saccharomyces cerevisiae* (yeast) and *atf-4* in *C. elegans*. Overexpression of *GCN4* and *atf-4* extends lifespan in *yeast* and *C. elegans*, respectively (23, 24), whereas knockdown reduces lifespan in yeast (25, 26) and had no effect on lifespan in *C. elegans* (27). Several more studies have found that knockdown of *atf-4* removed lifespan extension conferred by other genetic or pharmacological interventions (Table 1). In vertebrates, *ATF4* has been indirectly linked to aging and age-related phenotypes (Table 2). Strikingly, elevated levels of *ATF4* in liver and fibroblasts are a feature of many long-lived mice strains (28, 29), and such features have been argued to be highly informative of the biology of aging (30). However, some experimental evidence suggests the opposite; muscle specific knockout of *ATF4* protects against muscle aging in mice (31), and overexpression of *ATF4* in zebra fish causes early onset of hyperlipidemia (32).

**Table 1.** Lifespan effects of genetic manipulation of *ATF4* orthologs

Species	Gene name	Direction of manipulation	Effect on lifespan	Reference
*C. elegans*	*atf-4*	RNAi, Knockdown	No effect on lifespan	(27)
*C. elegans*	*atf-4*	Overexpression	Increased lifespan	(24)
*C. elegans*	*atf-4*	Knockdown	Loss of lifespan extension by *impt-1*	(33)
*C. elegans*	*atf-4*	Knockdown	Loss of lifespan extension by *mrps-5* RNAi	(34)
*C. elegans*	*atf-4*	Knockdown	Loss of lifespan extension by zidovudine	(35)
*C. elegans &* yeast	*atf-4/GCN4*	Knockdown	Loss of (replicative) lifespan extension by tRNA synthetase inhibitors borrelidin and mupirocin	(36)
yeast	*GCN4*	Overexpression	Increased chronological and replicative lifespan	(23)
yeast	*GCN4*	Knockdown	Reduced chronological lifespan	(25)
yeast	*GCN4*	Knockdown	Loss of replicative lifespan extension by depletion of 60S ribosomal subunits	(26)

This table lists all published studies in which *ATF4* or one of its orthologs was genetically manipulated (e.g., overexpression or knockdown) and reported effects on whole-organism lifespan. Only yeast and *C. elegans* studies are available. Note *atf-4* is referred to as *atf-5* in literature published before 2022, *GCN4* is the yeast *ATF4* ortholog.

**Table 2.** *ATF4* is linked to several age-related phenotypes across species

Species	*ATF4* ↑/↓	Observed Phenotype	Reference
*M. musculus and D. melanogaster*	**↓**	*ATF4* knockdown alleviates diet-induced diabetes, hyperlipidemia, and hepatosteatosis	(37)
*M. musculus*	**↑**	Elevation of *ATF4* in liver and fibroblasts is a common feature in many long-lived mice mutants	(28, 29)
*M. musculus*	**↓**	Tissue specific knockdown of *ATF4* in the liver results in increased oxidative stress and increased free cholesterol	(38)
*Danio rerio*	**↑**	Conditional *ATF4* overexpression promotes early onset of hyperlipidemia and enhances adipogenesis in zebrafish	(32)
*M. musculus*	**↓**	Tissue specific knockout of *ATF4* protects mice from common features of muscle aging	(30)
*M. musculus*	**↓**	*ATF4* drives regulatory T cell functional specification in homeostasis and obesity	(39)

↑Indicates upregulation of *ATF4*, ↓Indicates downregulation of *ATF4* either naturally, or as the result of genetic manipulation.

In addition to genetic manipulation, pharmacological interventions have also been shown to manipulate the GCN2–ATF4 pathway (40). Notably, treatment with tRNA synthetase inhibitors has been shown to activate the GCN2–ATF4 pathway (41–43). Interestingly, treatment with borrelidin (threonine tRNA synthetase inhibitor) and mupirocin (isoleucyl tRNA synthetase inhibitor) has recently been suggested to extend lifespan in an *ATF4*-dependent manner in yeast and *C. elegans* (36). Mechanistically, these inhibitors function by competing with their natural AA substrate, preventing proper charging of tRNAs. This process causes an accumulation of uncharged tRNAs, a hallmark of AA deprivation and a known trigger for GCN2 activation and subsequent upregulation of *ATF4* translation (44–46).

The direction of associations of aging with *ATF4* is variable (Tables 1 and 2). Still, the prevailing hypothesis, currently based on work in yeast and *C. elegans* and some associations in mice, combined with the idea that low stress activation is prolongevity (3), is that *ATF4* activation would lead to lifespan extension. There is however a lack of further experimental testing of the ISR pathway in aging research, especially in physiologically more complex and longer lived organisms than yeast and *C. elegans*. In this study, we conditionally manipulated the ISR pathway in flies (*Drosophila melanogaster*) via both *dGCN2* and its downstream target, *cryptocephal* (*crc*; Drosophila ortholog of *ATF4*, hereafter referred to as *dATF4*) to determine their role in aging. Experiments were conducted at fully fed and dietary restricted (DR) conditions, as mild stress and *ATF4* specifically have been implicated in the health benefits of DR (3, 47). We find contrary to the prevailing hypothesis that overexpression of *dATF4* shortened lifespan, whereas suppression of *dATF4* led to a robust lifespan extension.

## Results

### Suppression, Not Activation, of the GCN2–ATF4 Pathway Extends Lifespan.

**Fig. 1.**
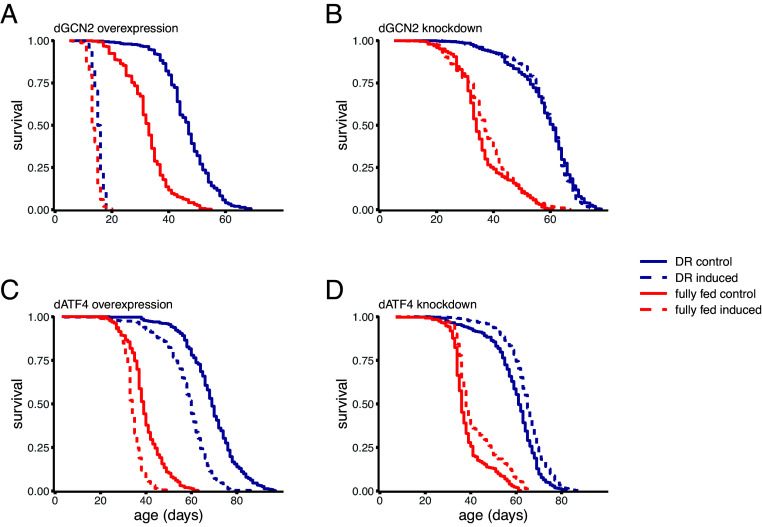
Lifespan is shortened by activation and extended by suppression of the GCN2–ATF4 pathway. Survival curves of flies with conditional induction of overexpression or knockdown (in vivo RNAi) of *dGCN2* (*A* and *B*) and *dATF4* (*C* and *D*) using *daGS* driver. All sample sizes and statistics are in *SI Appendix*, Table S1.

To isolate effects during adult life, we used a conditional driver to overexpress or knockdown *dGCN2* and its downstream effector *dATF4* during adulthood. A key benefit of using conditional drivers is that it fully controls for small genetic differences known to impact aging and lifespan (48, 49). Ubiquitous overexpression of *dGCN2* during adult lifespan drastically reduced lifespan (*P* < 0.0001, *N* ≥ 354; Fig. 1*A* and *SI Appendix*, Table S1). *dGCN2* overexpression effects were significantly stronger on DR (*P* < 0.01, *SI Appendix*, Table S1D). However, we interpret this as individuals dying rapidly irrespective of diet, with this effect appearing bigger on DR, as controls live longer on DR (*P* < 0.0001, *SI Appendix*, Table S1C). In line with the *dGCN2* overexpression results, reduction in lifespan was also observed when *dATF4* was overexpressed (*P* < 0.0001, *N* ≥ 376; Fig. 1*C* and *SI Appendix*, Table S1 A and B), independently of diet (*P* > 0.05, *SI Appendix*, Table S1D). Knockdown of *dGCN2* resulted in a nonsignificant lifespan extension (*P* = 0.06, *N* ≥ 391; Fig. 1*C* and *SI Appendix*, Table S1 A and B). Strikingly, when we knocked down *dATF4*, this significantly extended lifespan (*P* < 0.0001, *N* ≥ 363; Fig. 1*D* and *SI Appendix*, Table S2 A and B) irrespective of diet (*P* > 0.05, *SI Appendix*, Table S1D).

### Conditional Changes in dATF4 Transcript Levels Correspond with Reporter Activity.

**Fig. 2.**
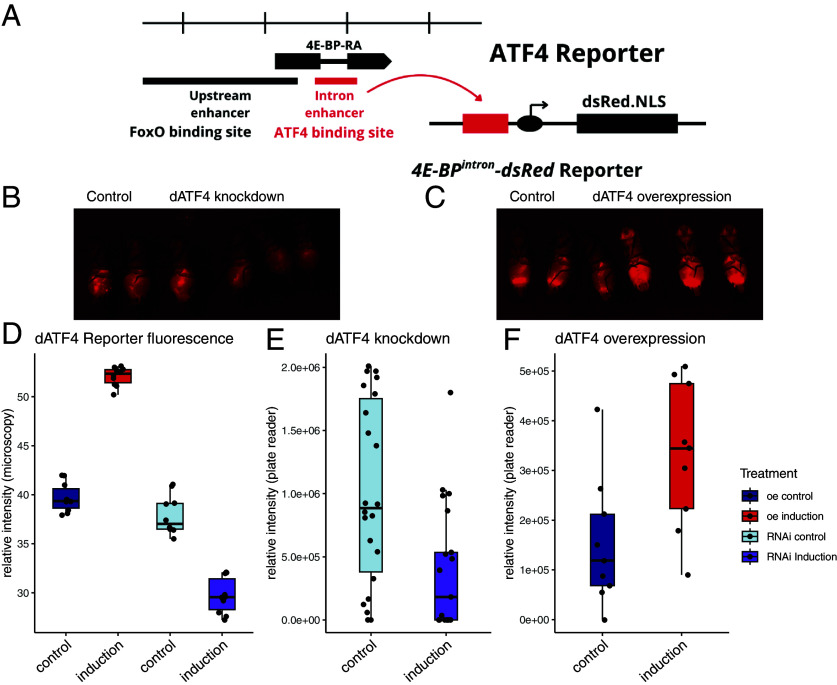
Conditional changes in *dATF4* transcript levels correspond with reporter activity (50). (*A*) Structure of fluorescent dATF4 reporter *4E-BP^intron^-dsRed* (Adapted from ref. 18). (B and *C*) Representative images of conditional induction vs. control of (*B*) *dATF4* overexpression and (*C*) *dATF4* knockdown on a fully fed diet. (*D*–*F*) Quantification of relative *4E-BP^intron^-dsRed* fluorescence (*D*) in the whole fly from microscopy images using pixel intensity measurements in ImageJ and (*E* and *F*) from the body using fluorescent spectrophotometry. RFU resulting from (*E*) 50 flashes and (*F*) 10 flashes.

As our results directly contradict prior findings on how *ATF4* modulates lifespan (Table 1), we wanted to confirm that in our experiments conditional changes in *dATF4* expression effectively altered downstream promoter activity. dATF4 activity can be visualized using a fluorescent reporter, *4E-BP^intron^-dsRed* (18, 50). As ATF4 is known to be highly regulated downstream of transcriptional regulation, both at the level of translation (51–53) and through posttranslational modifications (22, 54, 55), using an in vivo reporter is critical. The reporter contains an intron sequence with reported *dATF4* binding sites (Fig. 2*A*). Changes in dATF4 activity were visible already after 48 h after conditional induction of knockdown and overexpression (Fig. 2 *B* and *C*). Induction of *dATF4* overexpression increased reporter activity (*P* < 0.001, ANOVA, microscopy, Fig. 2*D*; *P* < 0.05, Welch *t* test, plate reader, Fig. 2*F*). A reduction in reporter activity was observed when *dATF4* was knocked down (Fig. 2*B*, *P* < 0.01, ANOVA, microscopy, Fig. 2*D*; *P* < 0.05, Welch *t* test, plate reader, Fig. 2*E*). dATF4 activity increased when *dGCN2* was overexpressed (*SI Appendix*, Fig. S1 *A* and *B*, *P* < 0.0001, Welch *t* test, microscopy). We validated *dATF4* knockdown using qPCR which led to a 1.5-fold reduction of *dATF4* expression (*SI Appendix*, Fig. S1*C*).

### Intensity and Cellular Context of GCN2–ATF4 Pathway Manipulation Modulate Lifespan Effects.

**Fig. 3.**
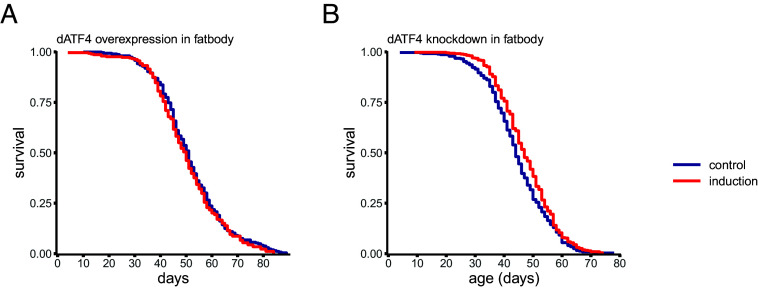
Lifespan extension resulting fro*m dATF4* knockdown, but not the reduction of lifespan seen when *dATF4* is overexpressed, is recapitulated in fat body specific context. Survival curves of flies with conditional (*A*) overexpression and (*B*) knockdown of *dATF4* using *S106GS* driver. All sample sizes and statistics are in *SI Appendix*, Table S3.

Intensity and cellular context of GCN2–ATF4 pathway activation are thought to be important in determining downstream target gene activation and ultimately cellular outcomes of ISR activation (22). To test this hypothesis, we used varying doses of RU486 supplementation to induce low, medium, and high levels of *dGCN2* and *dATF4* overexpression, respectively. Our results show a clear dose–response, but all manipulations reduced lifespan (*SI Appendix*, Fig. S2 and Table S2). We therefore have no evidence that a lower dose of *dGCN2* or *dATF4* overexpression could extend lifespan, but we cannot exclude that an even lower dose or tissue-specific manipulations could lead to a lifespan extension in response to overexpression. Indeed, effects of *ATF4* manipulation have been shown to vary by tissue (e.g., ref. 38) and cellular context of GCN2–ATF4 pathway manipulation, particularly the liver is thought to be an important regulator of several age-related phenotypes (Table 2). We tested the hypothesis that tissue-specificity may be important in determining lifespan outcome using *S106GS*, a GeneSwitch driver predominantly active in the fly abdominal fat body (analogous to liver and adipose tissue) (56, 57) to conditionally drive *dATF4* overexpression and *dATF4* knockdown. We find that in a fat body-specific context *dATF4* overexpression has no significant effects on lifespan (*P* = 0.278, *N* ≥ 298; Fig. 3*A* and *SI Appendix*, Table S3). Interestingly, we do recapitulate the lifespan extension we observed following ubiquitous *dATF4* knockdown, in a fat body-specific context (*P* = 0.018, *N* ≥ 380; Fig. 3*B* and *SI Appendix*, Table S3).

### Borrelidin Modulates Lifespan Effects of dATF4 Overexpression and Knockdown.

**Fig. 4.**
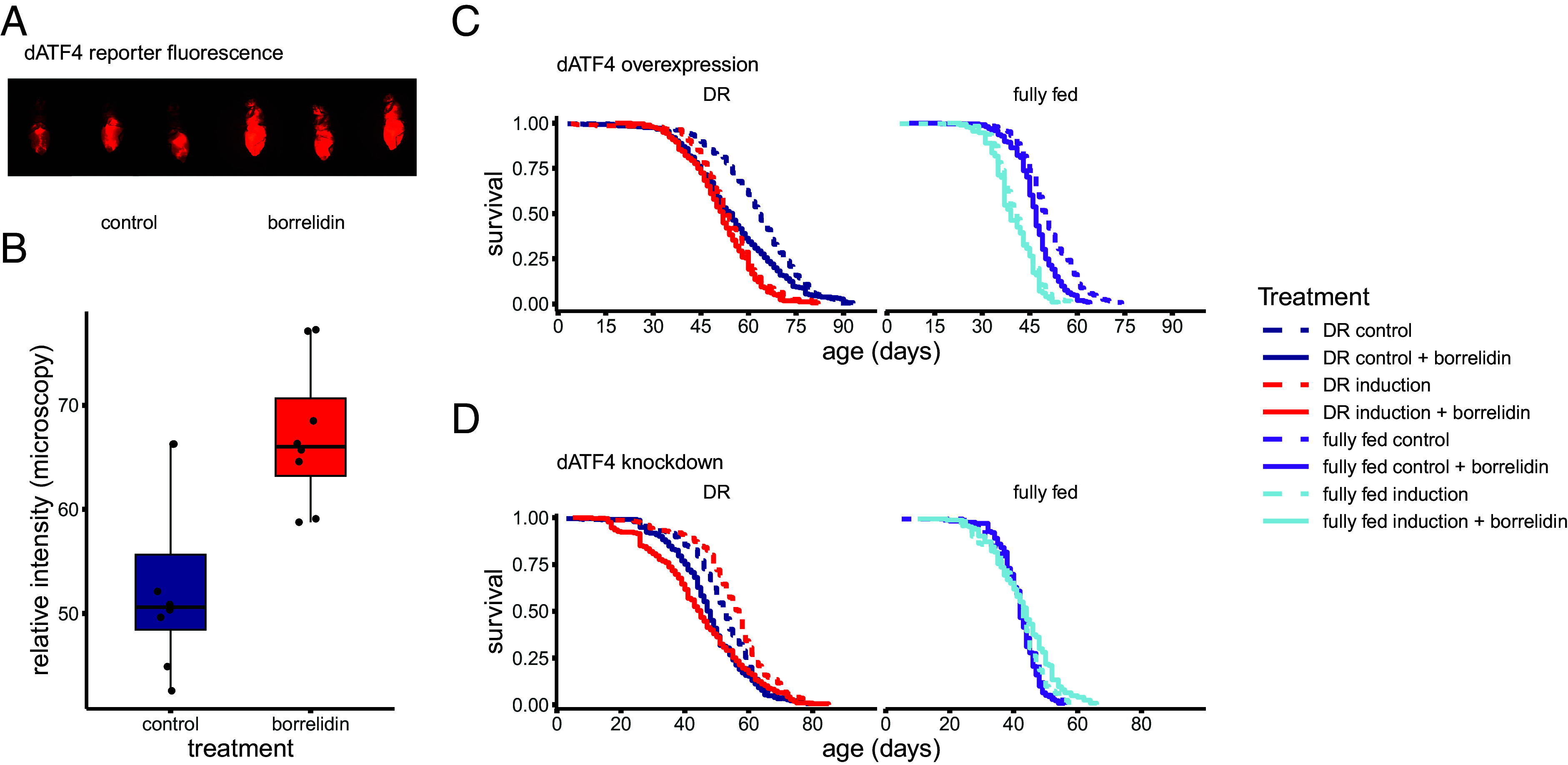
Borrelidin increases *dATF4* activity (*A* and *B*), modulates the lifespan effects of *dATF4* and reduces lifespan (*C* and *D*). (*A*) Representative images of changes in *dATF4* activity following 10 d on a fully fed diet supplemented with borrelidin compared to control. (*B*) Quantification of relative *4E-BP^intron^-dsRed* fluorescence in the whole fly from microscopy images using pixel intensity measurements in ImageJ. (*C*) Borrelidin did not reduce lifespan when *dATF4* was overexpressed. (*D*) Conversely, *dATF4* knockdown failed to extend lifespan when flies were treated with borrelidin. All sample sizes and statistics in *SI Appendix*, Table S4. Note sample sizes for fully fed conditions are lower especially for the *dATF4* knockdown condition (*B*, *Right*), and these results should therefore be considered less informative.

Pharmacological activation of the GCN2–ATF4 pathway, through tRNA synthetase inhibitors (specifically borrelidin), has recently been linked to lifespan extension in an *ATF4* and dose-dependent manner (36). In contrast to these findings, borrelidin reduced lifespan in flies (Fig. 4 and *SI Appendix*, Table S4), in line with the lifespan reduction we observed when *dATF4* is overexpressed. Note that dose is important here and we cannot exclude that at a lower dose or shorter exposure borrelidin can extend lifespan in the fly, as previous findings in other organisms showed a strong dose–response where higher doses greatly shortened lifespan while lower doses greatly extended it (36). Intriguingly borrelidin did not decrease lifespan further when *dATF4* was overexpressed (Fig. 4*A* and *SI Appendix*, Table S4C) and removed the lifespan extending effect of *dATF4* suppression (Fig. 4*B* and *SI Appendix*, Table S4D). We confirmed that borrelidin treatment significantly increases *dATF4* reporter activity (Fig. 4 *A* and *B*, *P* < 0.01, Welch *t* test). This in combination with the interaction between borrelidin and genetic manipulation of *dATF4* expression levels in our experiments does indicate that our genetic modification modulates the ISR effectively.

### Tyr Extends Lifespan but Does Not Affect Lifespan Extension by dATF4 Suppression.

**Fig. 5.**
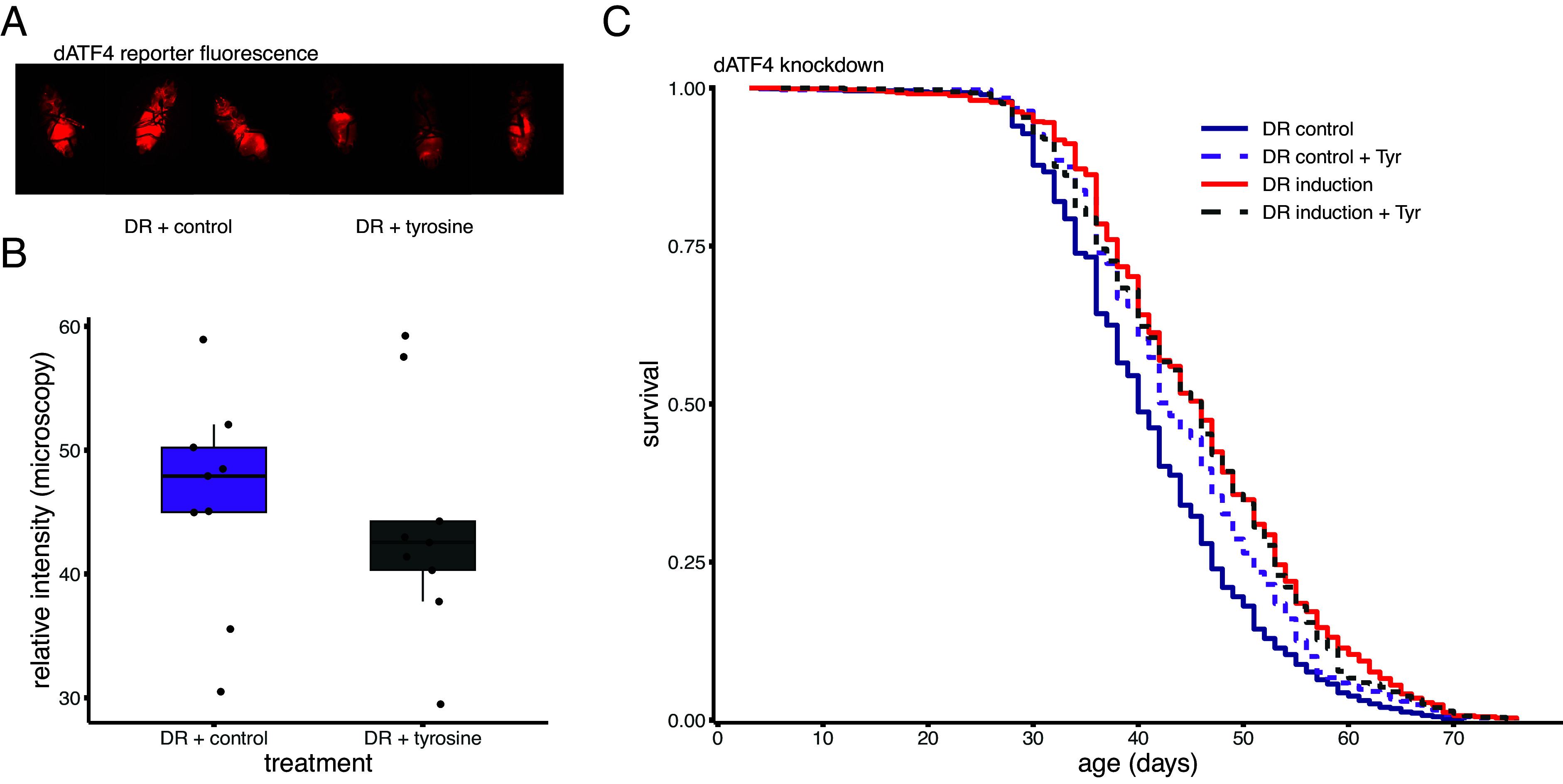
Tyr supplementation on a restricted diet extends lifespan in controls but not when *dATF4* is knocked down (in vivo RNAi). (*A*) Representative images in *dATF4* activity following 10 d on a DR diet supplemented with 5 mM Tyr compared to control. (*B*) Quantification of relative *4E-BP^intron^-dsRed* fluorescence in the whole fly from microscopy images using pixel intensity measurements in ImageJ showing a small but nonsignificant reduction in *dATF4* activity. (*C*) Survival curves of flies on a DR diet supplemented with either 5 mM Tyr or control in *dATF4* knockdown flies compared to controls. All sample sizes and statistics in *SI Appendix*, Table S5.

Restriction of Tyr has been shown to activate *ATF4* independently of GCN2 (18, 19). To confirm that the observed lifespan extension following *dATF4* knockdown, specifically under DR conditions was not the result of Tyr restriction, we supplemented our DR diets with 5 mM Tyr (19). The supplementation of Tyr in *dATF4* knockdown flies had no significant effect on lifespan (*P* > 0.62, *N* ≥ 626; Fig. 5*C* and *SI Appendix*, Table S5). Interestingly, we found that Tyr supplementation extends lifespan in control flies (*P* = 0.016, *SI Appendix*, Table S5). This suggests that dATF4 may be activated under DR conditions and that this activation is partially alleviated by Tyr supplementation (18). In this respect, we tested the effect of Tyr supplementation on *dATF4* activity. While we find that Tyr supplementation slightly reduced *dATF4* reporter activity, these changes were nonsignificant (Fig. 5 *A* and *B*; *P* = 0.64 Welch *t* test).

### Axenic Conditions Do Not Interact with the Lifespan Modulation of dATF4.

The described biological actions of borrelidin include antimicrobial effects (58). In addition, *ATF4* has been implicated in immunity (39, 59). To confirm that any observed changes in lifespan following borrelidin could be attributed to tRNA synthetase inhibition rather than its antibiotic properties, we treated conditional *dATF4* overexpression and knockdown flies with a broad range antibiotics to eliminate their microbiome. Antibiotics treatment had no significant effect on lifespan in either *dATF4* overexpression or knockdown flies (*P* > 0.33, *SI Appendix*, Fig. S3 and Table S6, respectively) and did not modulate lifespan changes observed via genetic manipulation of *dATF4* (*P* > 0.70, *SI Appendix*, Fig. S3 and Table S6). These results suggest that the observed lifespan changes are driven by borrelidin’s effect on the GCN2–ATF4 pathway, rather than its antibiotic properties or effects of *ATF4* on immunity which would likely lead to a differential response in axenic conditions.

### Transcriptomic Response to dATF4 Overexpression and Knockdown.

**Fig. 6.**
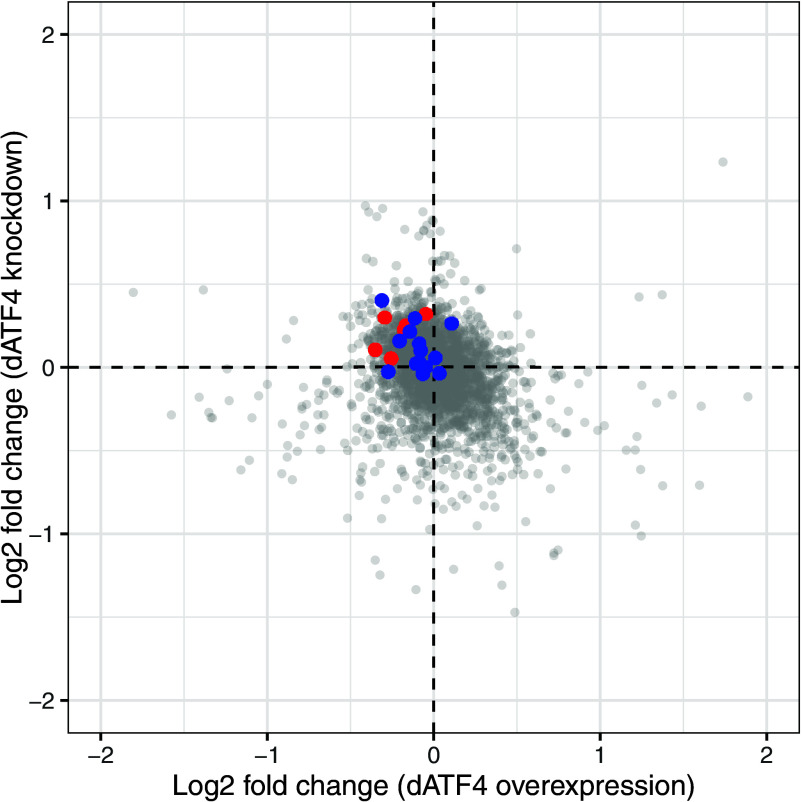
Rank correlation (−0.28, *P* < 0.0001) between differential expression in response to *dATF4* overexpression and knockdown suggesting concordant transcriptional change in response to *dATF4* directional manipulation. Highlighted genes from two concordant enrichment categories are highlighted, DNA mismatch repair (red) and Nucleotide excision repair (blue).

To further validate whether dATF4 modulation affected the transcriptome in opposite directions, we conducted ONT Transcriptome Sequencing. We measured the transcriptional response of whole flies 10 d after induction of *dATF4* overexpression or knockdown in comparison to their respective controls. Similar to the lifespan experiments, flies were kept either on fully fed or DR conditions. Given the lack of consistent diet-by-genotype interactions on lifespan (*SI Appendix*, Fig. S4 and Tables S1D and S7), we focused on transcriptomic differences driven by transgene induction alone. Samples were collected from both diets and an additive model controlled for the effect of diet while extracting the effect of transgene induction alone (*Methods*). We observed a modest, but statistically significant negative correlation in differential gene expression in response to *dATF4* knockdown and *dATF4* overexpression, suggesting *ATF4* targets are effectively modulated in our experiments (Fig. 6). Furthermore, when we selected genes that changed significantly in expression (*P* < 0.01, not FDR corrected), we found 18 genes that changed in both conditions, and of these, 13 changed in opposite directions. Although this overlap is not larger than expected by chance (*P* > 0.05) several of these genes have been previously reported to respond to *ATF4* across species, and their observed directional change in transcription matches with the predicted response in the literature (*SI Appendix*, Table S8), most notably *MAT2A* and *SLC36A1*. As such the analysis of significant gene overlap appears to support the biological conclusion that our experimental manipulation of *ATF4* levels changes the transcriptome in an ATF4-dependent manner as intended.

To explore the possible molecular mechanisms underlying the observed lifespan extension and reduction following conditional *dATF4* manipulation KEGG and GO-based gene set enrichment analysis was performed. We show the top 10 enriched up and down regulated terms irrespective of false discovery rate. This revealed distinct transcriptomic profiles for *dATF4* overexpression and knockdown, respectively; however, interestingly, we found that two DNA repair pathways (Mismatch repair and Nucleotide excision repair) were enriched in opposite directions, i.e., enriched in *dATF4* knockdown and suppressed in *dATF4* overexpression (Fig. 6). Overexpression of *dATF4* (*SI Appendix*, Fig. S4*A*) resulted in downregulation of DNA replication and mismatch repair and nucleotide excision repair, possibly indicating a breakdown of cellular maintenance. Furthermore, several metabolic pathways are downregulated, suggesting a shift away from carbohydrate metabolism. In addition, we observe an upregulation of glutathione metabolism, a pathway involved in detoxification processes, often associated with oxidative stress, and aminoacyl-tRNA biosynthesis and Glycine, serine and threonine metabolism, possibly reflecting stress-related changes in protein homeostasis. Intriguingly, further GO term analysis suggested reduced capacity to respond to stress (*SI Appendix*, Fig. S4*B*). Knockdown of *dATF4* changed pathways associated with energy metabolism, protein synthesis, and cellular maintenance (*SI Appendix*, Fig. S4*C*). Oxidative phosphorylation and the citrate cycle (TCA cycle) were downregulated, which suggests reduced energy expenditure. Alongside this, AA biosynthesis and ribosome-related pathways were also downregulated, suggesting a broader suppression of anabolic activity and translation. In combination, these changes may reflect a shift away from energy-expensive processes and a reduction in cellular workload.

### dATF4 Lifespan Effect Is Consistent across Three Replicates.

To analyze the consistency of *dATF4* manipulation on lifespan, we compared the primary *dATF4* lifespan results (Fig. 1) to a reanalysis of the control groups of two independent experiments (Fig. 3 and *SI Appendix*, Fig. S1). These replicates were conducted in the same lab, but at different times and differ only in sample size and vehicle control added to the food (*Methods*). Overexpression of *dATF4* reduced lifespan across all experiments (*SI Appendix*, Table S4C). In one experiment, the effect of overexpression on lifespan was smaller on DR. Similarly, *dATF4* knockdown extended lifespan in all experiments. In one experiment effects are again weaker under DR, but at this moment, we have no strong evidence that the effects of *dATF4* on lifespan are diet-dependent. When we combine all three experiments in a single statistical model, we find no evidence for an interaction between knockdown of *dATF4* and diet (logHR = −0.19 ± 0.13, *P* = 0.13), but some evidence for an increase in mortality through *dATF4* overexpression on DR (logHR= 0.44 ± 0.22, *P* = 0.046). The effects of *dATF4* on lifespan are remarkably similar, making us confident that its effect on lifespan we report is robust.

## Discussion

Overexpression of *ATF4* orthologs in yeast and *C. elegans* has been associated with lifespan extension. In contrast, our work shows that activation of the GCN2–ATF4 pathway, via borrelidin, overexpression of *dGCN2* and its downstream effector *dATF4*, reduces lifespan. However, when expressed in a tissue-specific manner in the fat body, no lifespan shortening effect of *dATF4* was found. *ATF4* remains an important geroscience target as *dATF4* knockdown resulted in a robust lifespan increase, when applied in the whole fly or in the fat body specifically. These findings highlight the GCN2–ATF4 pathway as a modifiable regulator of lifespan in flies, with potential cross-species relevance.

Exposure to mild stress and the cellular stress responses they activate are generally thought to extend lifespan (4, 60–63). Overexpression of stress-responsive transcription factors is generally associated with increased longevity, most clearly and repeatedly demonstrated in *C. elegans* (64–68). ATF4 has been proposed to play a similar prolongevity role (Table 1). However, our work shows that conditional systemic overexpression of *dATF4* reduces lifespan in flies, challenging the prevailing interpretation that *ATF4* is universally beneficial and suggesting a more nuanced interpretation of ATF4’s role in geroscience. One explanation for the contradictory findings across studies may lie in the complex and context-dependent nature of ATF4 signaling. Low level or short term activation of *ATF4* leads to upregulation of prosurvival pathways (21, 69); *ATF4* activation at high levels or for an extended period of time may result in the upregulation of pro-apoptotic pathways (20, 70–73). We show that while negative lifespan effects of GCN2–ATF4 pathway activation are dose responsive, at all tested doses, we find a reduction of lifespan, suggesting our manipulation may still be too severe to observe lifespan extending effects of *ATF4*. This supports a model in which chronic or intense *ATF4* activation leads to maladaptive effects and aligns with a growing body of evidence that *ATF4* activation effects are multifaceted (37, 39, 74, 75).

Other studies, again mainly in *C. elegans* suggest that the relationship between stress pathway activation and lifespan is context-dependent and may also depend on the tissue or timing of expression. For example, constitutive overexpression of *ATFS-1* has been shown to reduce lifespan (76, 77) and constitutive overexpression of *XBP-1* has tissue-specific effects on lifespan. Ubiquitous and muscle-specific overexpression of *XBP-1* reduces lifespan, while tissue-specific overexpression in neurons or the intestine extends lifespan (78). Indeed, effects of *ATF4* were suggested to potentially be tissue-specific (38). This aligns with our finding that fat body-specific *dATF4* overexpression has no effect on lifespan. Perhaps the negative effects on lifespan we see when *dATF4* is overexpressed in the whole fly are due to expression in tissues which would normally not respond to (prolongevity) mild stress by activating *ATF4*. Thus, even though *ATF4* activation remains a highly promising geroscience strategy in vertebrates (28, 29), our results suggest benefits may be context, tissue, and dose dependent.

Indeed, reported lifespan effects of the ISR are variable, with manipulation of some pathway genes (e.g., *eIF2Bγ/ppp-1* and *eIF2αS51A;* 79) suggesting that suppression rather than activation of the ISR promotes longevity (11). Effects of *ATF4* knockdown on lifespan were shown to be negative in yeast or have no effect in *C. elegans* (Table 1). Intriguingly, we find that *dATF4* knockdown in flies extends lifespan in both systemic and fat body-specific conditions. This fits with some reports in mice; *ATF4* knockout mice are protected from age-related muscle degeneration (31). Furthermore, *ATF4* activation is frequently hijacked by tumor cells to enhance cell survival from the stress that results from rapid proliferation and nutrient limitation (80, 81). In contrast, increased *ATF4* activity was found to sensitize tumor cells to therapy-induced cell death (73, 82). This highlights that *ATF4*-driven stress responses may confer either resilience or vulnerability, depending on the context (54). Our findings therefore suggest a more nuanced interpretation of *ATF4*’s role in the biology of aging. Testing whether *ATF4* activation or suppression are similarly context dependent in conferring healthspan benefits in vertebrates will be a key next step in understanding how we harness *ATF4*’s biology in geroscience, as well as understanding the molecular biology of *ATF4* and its upstream regulation and downstream physiological targets.

*ATF4* activation is (in part) regulated upstream through autophosphorylation of GCN2 by sensing of uncharged tRNAs, and subsequent phosphorylation of eIF2α (14). Interestingly, two types of tRNA synthetase inhibitors, including borrelidin, are thought to extend lifespan in yeast and *C. elegans* in an *ATF4*- and dose-dependent manner (Table 1). Additionally, other work in both *C. elegans* (83, 84) and flies (85) has shown that RNA interference (RNAi) of transfer RNA (tRNA) synthetases is associated with lifespan extension. Borrelidin, a known tRNA synthetase inhibitor, activates the GCN2–ATF4 pathway via accumulation of uncharged tRNAs, thereby mimicking AA deprivation (86). This positions borrelidin as a mechanistic probe of the GCN2–ATF4 pathway. However, we find that, at least at our tested dose, borrelidin significantly reduces lifespan. Consistent with our findings that overexpression of *dGCN2* and its downstream target *dATF4* reduces lifespan, we find that pharmacological activation of this pathway using borrelidin reduces lifespan in an *dATF4*-dependent manner. We find that borrelidin does not reduce lifespan when *dATF4* is overexpressed and that borrelidin removes the lifespan extension resulting from *dATF4* knockdown. Perhaps overexpression of *dATF4* is already exerting its maximum negative effect on lifespan and additional pharmacological activation by borrelidin is therefore not observed. However, in this scenario, we would expect knockdown of *dATF4* to rescue the negative effects activation of *dATF4* by borrelidin has on lifespan. This is not the case, as on DR, knockdown of *dATF4* exacerbates the lifespan shortening by borrelidin, suggesting more complex regulation of *ATF4*s targets other than through *ATF4* abundance.

A possible explanation for these results is that the experimental modulation of *dATF4* transcript levels changes posttranslational modifications of *ATF4*. The relative posttranslational state of *ATF4* is understood to be complex and the reason why *ATF4* activation can both lead to suppression and activation of its target genes (22, 54, 55). Borrelidin may independently modulate *ATF4*’s posttranslational modifications through GCN2 and may therefore not lead to the expected directional relationships. As such our wider results could indicate that *dATF4* knockdown extends lifespan as it leads to a more prolongevity genomic transcriptional signature, perhaps not through the abundance of dATF4 protein, but its relative posttranslational state. This is an intriguing yet currently speculative hypothesis that will require further testing.

The downstream mechanisms that are responsible for the lifespan extension we see when *dATF4* is suppressed remain to be elucidated. The fly is an excellent model to investigate these mechanisms as both the diet and associated genetic pathways can be experimentally manipulated with relative ease. As prior work has suggested that tyrosine restriction can activate *ATF4* (18, 19), we tested the effects of tyrosine supplementation. Interestingly, tyrosine supplementation extended lifespan in controls under DR conditions, but had no effect in flies where *dATF4* was knocked down. Our work suggests that tyrosine metabolism is not part of the downstream mechanisms responsible for the lifespan extension induced by *dATF4* suppression. In contrast, tyrosine may suppress dATF4 activity and thus indirectly lead to a lifespan extension.

We conducted our analysis of the transcriptomic response to *dATF4* overexpression and knockdown with the intention of validating that our manipulations resulted in the intended opposite responses on the transcriptome. An added benefit is that these transcriptomes provide suggestions about the possible mechanisms involved, albeit correlative. Overexpression of *dATF4* resulted in a reduction of carbohydrate-based energy metabolism and DNA repair mechanisms, with an upregulation of detoxification pathways, including glutathione metabolism, which is important for responding to oxidative stress (87–89). The observed upregulation of aminoacyl-tRNA biosynthesis is consistent with findings that suggest tRNA synthetases may reflect a compensatory mechanism that is activated under chronic activation of the ISR (90) and could explain why further activation of the GCN2–ATF4 pathway via tRNA synthetase inhibition had limited additional effects on lifespan in *dATF4*-overexpressing flies. In combination, this transcriptional profile suggests a stress-induced reprioritization of transcriptional programs (91–93), which when chronically activated may be detrimental or predisposing cells to apoptosis (20, 54, 59, 70–73, 94). In contrast to the stress-associated signature of the overexpression data, we find that *dATF4* knockdown resulted in a downregulation of oxidative phosphorylation and the TCA cycle. This contrasts with previous findings in prolongevity contexts, which link upregulation of oxidative phosphorylation to lifespan extension in DR and long-lived mutant mice (95, 96). Notably, in a cortical neuron model, *ATF4* is thought to be a prodeath transcription factor with *ATF4* knockdown in cortical neurons thought to increase resistance to oxidative death (97). However, the overall suppression of anabolic pathways (including AA biosynthesis and ribosome-related pathways) is consistent with other prolongevity phenotypes (98). Furthermore, we found various DNA repair pathways enriched in *dATF4* knockdown flies. Although this may reflect a response to increased damage, it could also be a sign of increased cellular maintenance (99). Additionally, we observe upregulation of proteasome and ubiquitin-mediated proteolysis pathways, which contribute to cellular maintenance. Previous work on *C. elegans* suggests that upregulation of the proteasome in response to mitochondrial stress, potentially caused by a downregulation of oxidative phosphorylation, leads to lifespan extension (100). Together, these findings suggest that the prolongevity effect of *dATF4* knockdown may be the result of a coordinated shift toward cellular maintenance.

Our study challenges the prevailing hypothesis that *ATF4* activation is broadly beneficial for lifespan by demonstrating that, in flies, chronic or systemic activation of the GCN2–ATF4 pathway can be detrimental, while suppression of *dATF4* extends lifespan. Our findings suggest that the role of *ATF4* in aging is likely to be dose, tissue, and context-dependent. Our work further positions the GCN2–ATF4 pathway as a modifiable control of lifespan.

## Methods

### Fly Husbandry and Diets.

All flies were reared on “fully fed” fly media (8% yeast), prepared to the following specifications: 8% yeast, 13% table sugar, 6% cornmeal, 1% agar, and 0.225% nipagin (all w/v) (99, 101). In the case of growing bottles 0.4% (v/v) propanoic acid was also added. Cooked fly media were stored at 8 °C for up to 2 wk and were warmed before use. All experiments were done in a climate-controlled environment with a 12:12 h light–dark cycle, at 25 °C and ±60% humidity.

Experiments were conducted at two dietary conditions, 2% yeast (dietary restricted; DR) and 8% yeast (fully fed), with all other dietary components remaining the same. Where the GeneSwitch construct was used, food was supplemented with RU486 (Thermo Fisher Scientific) to induce transgene expression, or the same volume of absolute ethanol once the food cooled down (~80 °C), as (200 μM final media concentration; or 100 μM and 50 μM for dose–response; dissolved in 8.6 mL absolute ethanol (Fisher) per 1 L of fly media and mixed into the media) or control food lacking RU486 (but still containing 8.6 mL absolute ethanol per 1 L of fly media) (48, 102). Prior work by ourselves and others has not detected any effects of RU486 on longevity (48, 95, 102–105).

Where specified, 50 µL of 60 µM Borrelidin stock solution or a control (3% ethanol), was pipetted onto the experimental diet, for a final concentration of 3 µM Borrelidin, assuming even distribution within the top ~1 mL surface layer of food (106). To distinguish effects of borrelidin from its antimicrobial properties (58), we conducted a separate lifespan experiment where flies were treated with 50 µL of a broad-spectrum antibiotic cocktail (final concentration in the diet: 100 µg/mL ampicillin, 50 µg/mL vancomycin, 100 µg/mL neomycin, and 100 µg/mL metronidazole), or equal volume of dH_2_O for 10 d we previously validated eradicates the microbiome (98). To assess whether *dATF4* knockdown effects on DR were the result of Tyr restriction, we supplemented our DR diet with 1 g/L of Tyr, resulting in 5 mM final concentration of Tyr supplementation (19).

### Genotypes.

All fly genotypes used in this study are listed in Table 3.

**Table 3.** List of all fly genotypes used in this study and how they were obtained

Genotype	Purpose	Source/ Bloomington ID
*w;UAS-dGCN2-CA*	Males crossed with driver to overexpress *dGCN2*	P Leopold, University of Paris (107)
*yv;UAS-dGCN2-RNAi*	Males crossed with driver to knockdown *dGCN2*	TRiP, BDSC 67215
*w;;UAS-dATF4*	Males crossed with driver to overexpress *dATF4*	BDSC 81655 (108)
*yv;UAS-dATF4-RNAi*	Males crossed with driver to knockdown *dATF4*	TRiP, BDSC 80388
*w;;4E-BP^intron^-dsRed*	Fluorescent *dATF4* Activation Reporter	Obtained through Fumiaki Obata (50)
*yw;daGS*	Females crossed with UAS lines for conditional, ubiquitous overexpression/knockdown of gene of interest	Obtained through Marc Tatar (109)
*yw;S106GS*	Females crossed with UAS lines for conditional fat body-specific overexpression/knockdown of gene of interest	Obtained through Marc Tatar (57)

### Lifespan Experiments.

The main lifespan experiments were performed using females expressing the *daughterless* (*da*) GeneSwitch system (*daGS-GAL4*). The *da* promoter is active in almost all cells in the fly; using the GeneSwitch system therefore allows us to conditionally overexpress and knockdown (in vivo RNAi) genes in the whole fly (109). 3 to 5 males carrying various UAS transgenes (Table 3) were crossed to 10 to 12 virgin females in bottles to control for density. As F1 progeny eclosed they were transferred to new bottles each day to generate age-matched cohorts, and left to mate for 2 d. Mated offspring were anesthetized using carbon dioxide (Flystuff Flowbuddy; <5 L/min), and females of the desired genotype were sorted into groups of up to 100 individuals and transferred to custom built demography cages (previously described in refs. 100 and 110). Flies were switched to experimental diets at age 3 to 4 d. After caging, mortality was measured every other day by conducting a fly census. At this time point, food vials were replaced, and dead flies were counted and removed from cages. Escaped and accidentally killed flies, and those stuck on the diet were right-censored (i.e., removed from mortality analysis as their cause of death could not be attributed to the experimental conditions tested).

### Imaging and Quantification of dATF4 Activity.

A Leica M165 FC fluorescent dissection microscope was used to image flies expressing the *dATF4* reporter using dsRed filters, using LasX (Leica) image acquisition software. Adult females were used and kept on their respective diets for 2 d (*dATF4* overexpression, *dATF4* knockdown, and dGCN2 overexpression) and 10 d (borrelidin and tyrosine) before imaging. All experiments were conducted on rich diets, apart from the tyrosine addition experiments which were done on restricted diets as in the corresponding lifespan experiments. The following settings were used for dATF4 overexpression and dATF4 knockdown: exposure = 50 ms, gain = 3.0, zoom = 1.25; dGCN2 overexpression: exposure = 50 ms, gain = 3.0, zoom = 2.5; borrelidin and Tyr: exposure = 30 ms, gain = 3.0, zoom = 2.5. Fluorescence on images was quantified using Pixel Intensity Measurement in ImageJ. *dsRed* fluorescence of conditional *dATF4* overexpression and knockdown, was further quantified using fluorescent spectroscopy (*N* ≥ 10). Individual *dATF4* overexpression and *dATF4* knockdown flies were lysed with 5 mm stainless steel beads (Qiagen) in PBS (1,000 µL PBS and 500 µL PBS, respectively) for 3 min at 25 Hz using TissueLyser III (Qiagen). Samples were then centrifuged for 3 min at 13,000 rpm. The supernatant of samples was pipetted on 96-well, black-bottom cell culture microplates (Greiner BIO-ONE) and fluorescence was then measured using HIDEX Sense Beta Plus Microplate Reader (Type 425-311). The following settings were used: Technology = Fluorescence, Excitation = 560/40 nm, Emission = 590/20 nm, Mirror = Automatic, PMT voltage = 575 V, Flashes = 50 (*dATF4* knockdown)/10 (*dATF4* overexpression). The volume of PBS and the number of flashes used to analyze *dATF4* knockdown flies was adjusted to account for the expected reduction in reporter activity and ensure measurements remain in the linear detection range of signal intensity. The average fluorescence of two technical replicates was used.

### Data Analysis for Lifespan, Fecundity, and dATF4 Activity.

Data handling of lifespan data was done using a custom pipeline using spreadsheet software and R. Changes in lifespan associated with expression of different genes, dietary conditions, and pharmacological interventions were analyzed using the *coxme* package in R (111). The models accounted for cage effects (using a random effect) and shared days of sorting into cages (batch) using a fixed effect. Models that combined the three lifespan experiments, included experiment as a random term. Each sorting day flies were split across different dietary conditions thereby balancing treatments. Changes in *dATF4* activity were analyzed using ANOVA and post hoc *t* test to analyze imageJ results and Welch *t* test for plate reader results.

### RNA-Sequencing (RNA-seq) Library Preparation.

For RNA-seq analysis of *dATF4* overexpression and knockdown, flies on both dietary conditions (DR and fully fed) were sampled from cages after 10 d on RU486 or control diets. Samples were processed as a mix of 4 to 6 whole flies, snap frozen, and kept at −70 °C until analysis. Total RNA was extracted from 4 to 6 whole flies using Qiagen RNeasy Mini kits, following RNeasy Mini Kit Quick-Start Protocol. cDNA libraries for Oxford Nanopore sequencing were prepared using the cDNA-PCR Barcoding Kit V14 SQK-PCB114.24 kit (Oxford Nanopore Technologies; ONT), following cDNA-PCR Sequencing V14—Barcoding (SQK-PCB114.24) protocol with modifications. cDNA concentration was measured using Qubit and 1.925 ng barcoded cDNA per sample was added to each library. In total three libraries were prepared to contain two biological replicates per condition to make up a total of 16 samples per flow cell. A total of three flow cells were run providing a N = 6 per condition. Sequencing was done using GridION X5 (ONT).

### qPCR.

qPCR was used to quantify the knockdown of *dATF4*. 500 ng of the same RNA samples as used to for sequencing were reverse transcribed using the PrimeScript FAST RT reagent kit with gDNA eraser (Takara) and qPCR was undertaken using the TB Green Premix Ex Taq II FAST qPCR kit (Takara) and the 2^−ΔCT^ method was used to normalize results by expression of the housekeeping gene, *Act5C* (β-actin), as described previously (112). The primers used to amplify targets from cDNA are as follows (all shown 5’ to 3’): *Act5C* forward (ACACAAAGCCGCTCCATCAG), *Act5C* reverse (TGTCGACAACCAGAGCAGCA), *dATF4* forward (GTGATGAGGAGATGGTTGTGGAG), and *dATF4* reverse (TGTTTGCGGGTACTATGGCC).

### Transcriptomic Analysis.

For bioinformatic analysis, we used minimap2 (v2.24; 113) and Salmon (v1.10.2; 114), and we used the BDGP 6.32 genome and associated transcript annotation file as reference files. Prior to differential gene expression analysis, we filtered out libraries with less than 400,000 reads. This resulted in exclusion of two libraries in the *dATF4* overexpression dataset (DR control and fully fed control) and three libraries in the *dATF4* knockdown dataset (2 DR control and 1 DR induced). Low abundance genes were filtered out from the dataset to improve statistical power and minimize false positives. Genes were included in the analysis if they had a count-per-million (CPM) greater than 15 in all samples included in further analysis. The CPM threshold was selected based on exploratory data analysis to ensure reliable read-depth for reliable differential expression analysis, while maintaining sufficient gene representation. Differential expression analysis was done using the *edgeR* package and “glmFit” (115) using an additive model of diet and transgene induction. Rank correlation between *dATF4* overexpression and *dATF4* knockdown was determined using Spearman’s rank correlation coefficient. Gene set enrichment analysis (GSEA; 116) was performed to identify biological pathways and processes associated with the differential gene expression profile. The KEGG (Kyoto Encyclopedia of Genes and Genomes; 117) and GO (Gene Ontology; 118–120) pathway database was used as reference gene sets for enrichment testing.

## Supplementary Material

Appendix 01 (PDF)

## Data Availability

Study data are included in the article and/or *SI Appendix*.
