# Supplementary material for: Suppression rather than activation of the integrated stress response (GCN2–ATF4) pathway extends lifespan in the fly
Source: Proc Natl Acad Sci U S A. 2026 Apr 28;123(18):e2518812123. doi: 10.1073/pnas.2518812123 (PMC13142962; doi:10.1073/pnas.2518812123)
Supplement: Supplementary file 1 — Appendix 01 (PDF) [file pnas.2518812123.sapp.pdf]

Supplementary Information Appendix

Suppression rather than activation of the integrated-stress-response (GCN2-ATF4) pathway extends lifespan in the fly

Götz *et al.* 2026.

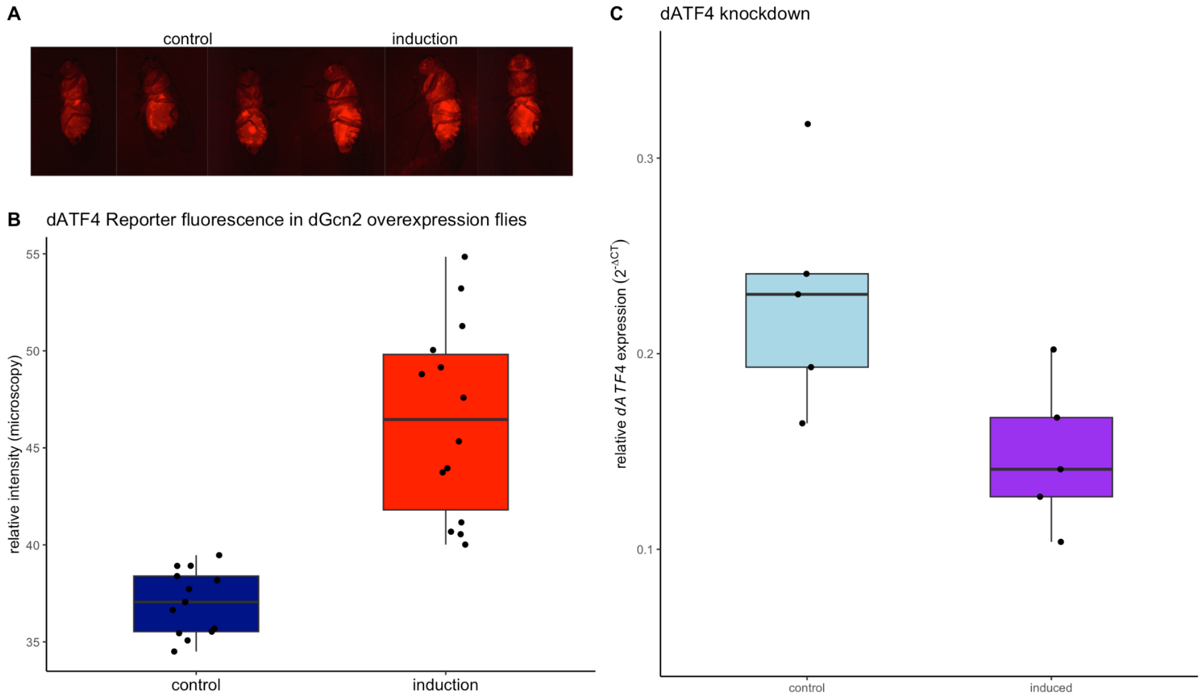

Figure S1. (A) Representative images of dATF4 activity in *dGCN2* overexpression flies under induction and control conditions. (B) Quantification of relative *4E-BP*^{intron}*-dsRed* fluorescence in the whole fly from microscopy images using pixel intensity measurements in ImageJ. (C) Quantification of relative *dATF4* expression in *dATF4* knockdown using qPCR in flies on DR (2% yeast).

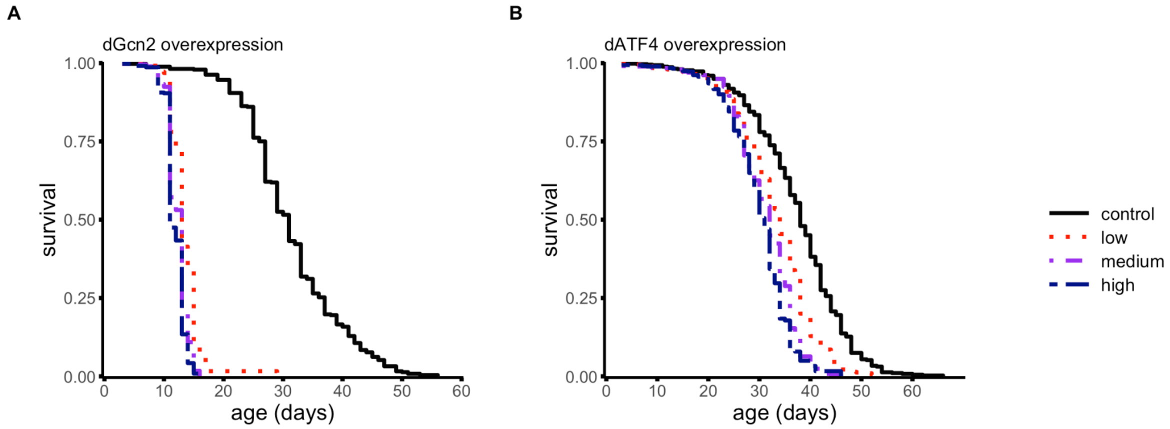

Figure S2. Lifespan reducing effects of *dGCN2* and *dATF4* overexpression are dose responsive. Survival curves of flies on a fully fed diet with low, medium and high levels of induction of (A) dGCN2 overexpression and (B) *dATF4* overexpression using different doses of RU486 (see methods). All sample sizes and statistics are given in Table S2.

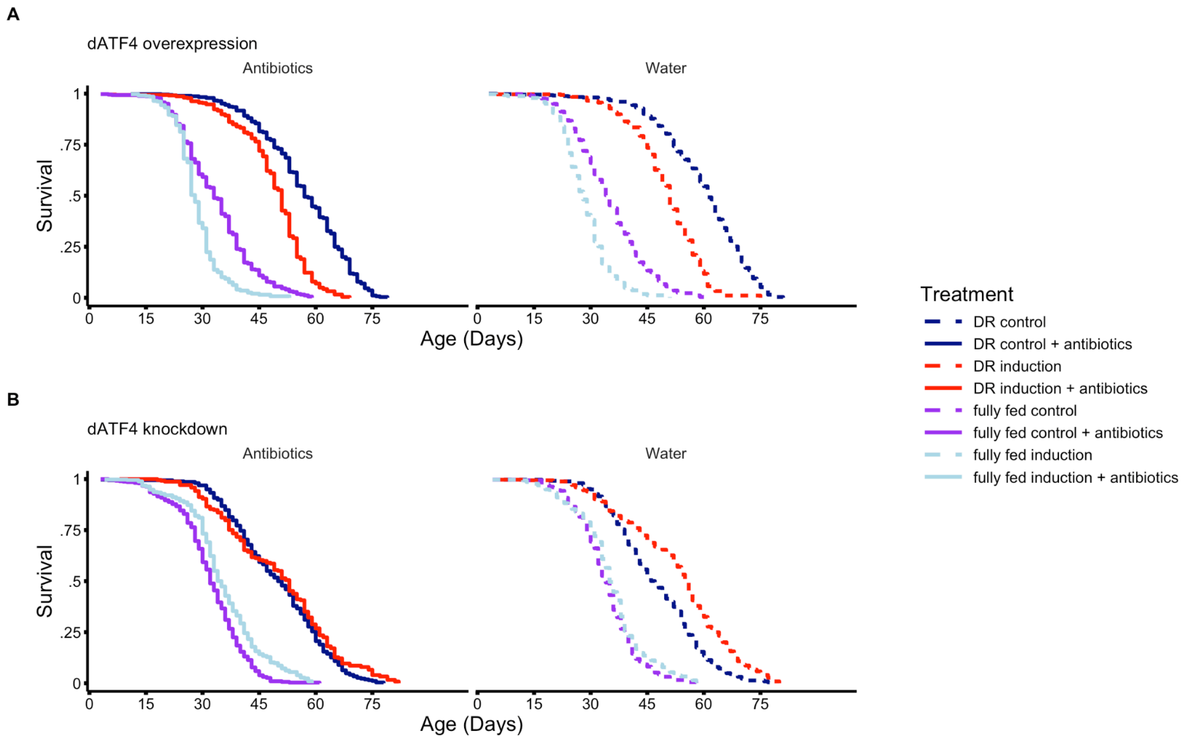

Figure S3. Microbial load does not affect the lifespan effects of *dATF4* manipulation. Survival curves of flies where the GeneSwitch construct was used to conditionally induce (A) overexpression or (B) knockdown (*in vivo* RNAi) of *dATF4* to test the effect of microbial load on the lifespan effect of *dATF4* manipulation. Flies on antibiotic treatment were treated with 50µl of broadband antibiotics to eradicate their microbiome (see methods), control flies were treated with the same volume of dH_2O. All sample sizes and statistics are given in Table S6.

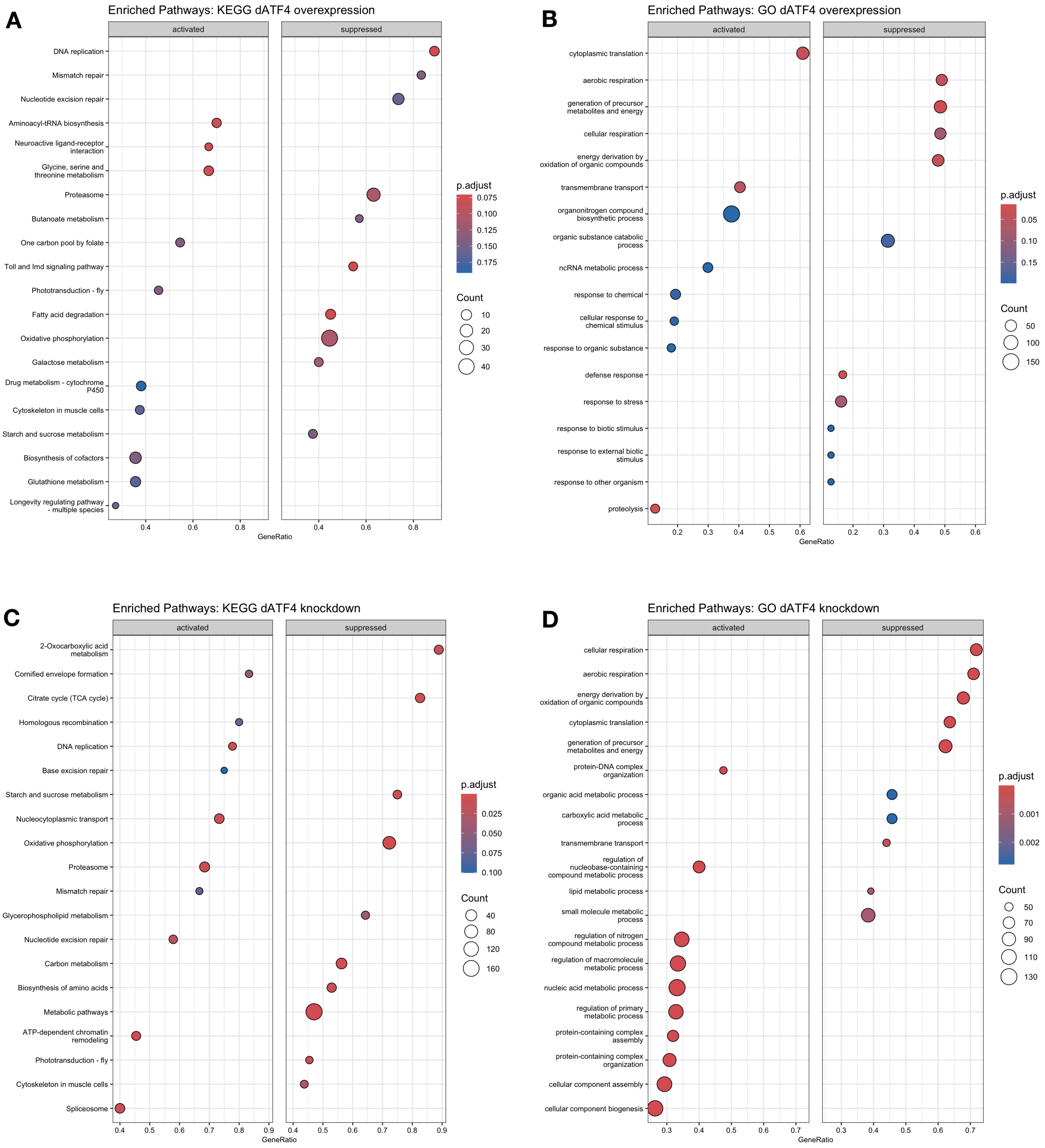

Figure S4. Top 10 activated and suppressed KEGG Gene Set Enrichment Analysis and GO terms, respectively for the effect of induction of *dATF4* overexpression and knockdown. The data are split for pathways that were activated or suppressed in (A,B) *dATF4* overexpression and (C,D) *dATF4* knockdown (*in vivo* RNAi).

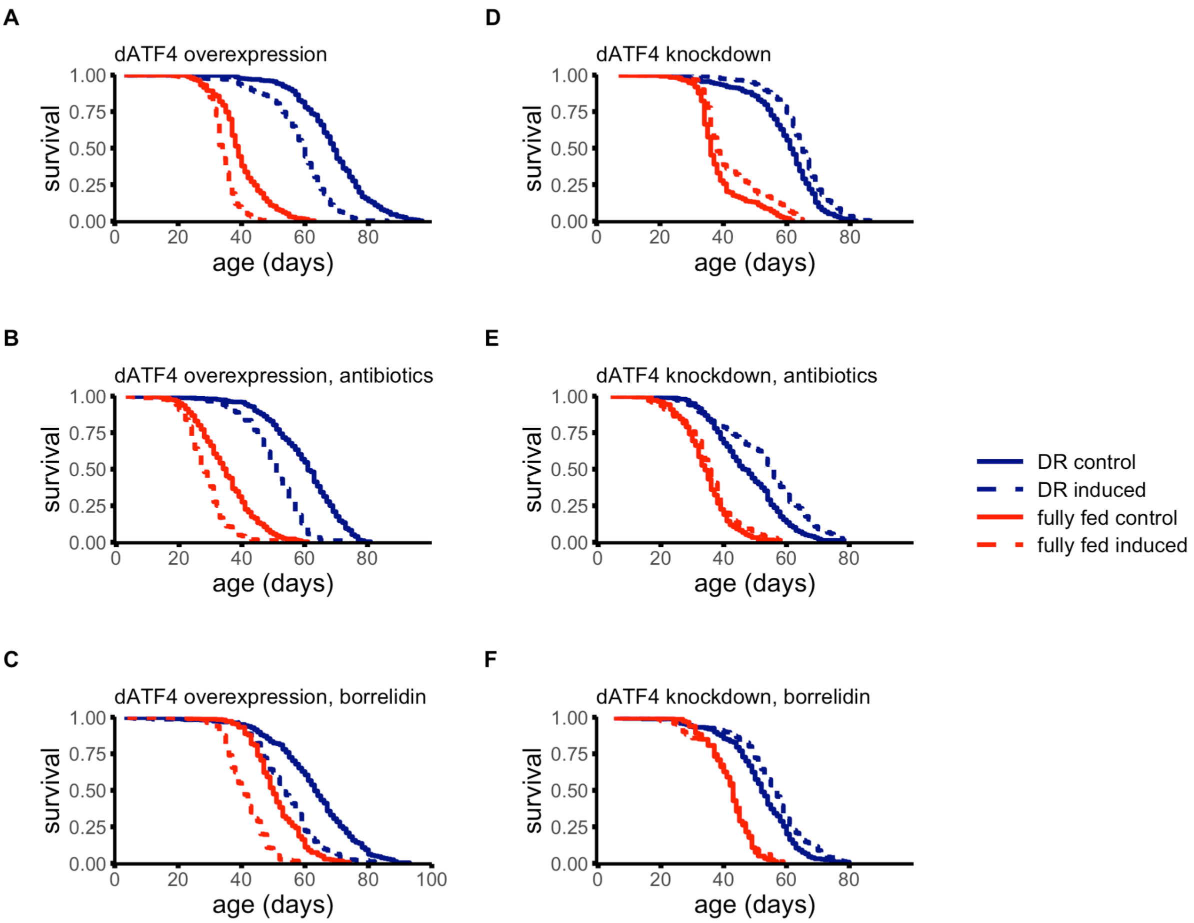

Figure S5. *dATF4* overexpression consistently reduces lifespan, while *dATF4* knockdown extends lifespan. Survival curves of flies where the GeneSwitch construct was used to conditionally induce (A-C) overexpression or (B-F) knockdown (*in vivo* RNAi) of *dATF4.* Replication was obtained from the control experiments for the “antibiotics” and “borrelidin” experiments and analysed separately. These controls contained an additional 50µl dH_2O or 3% ethanol respectively. All sample sizes and statistics are given in Table S7. Note, sample size of the knockdown fully fed group of dATF4 knockdown from the “borrelidin experiment” is low.

Table S1. Statistics for the effect of genetic manipulation of the GCN2–ATF4 pathway on lifespan. A) All sample sizes per treatment and condition. B-D) Statistics for effect of (B) conditional overexpression/knockdown of *dGCN2* and *dATF4*; (C) DR (2% yeast) in comparison to fully fed conditions (8% yeast); (D) the interaction of diet on gene induction on lifespan. B-D Statistics calculated using Coxme R package.

A)		Sample Sizes		
**Condition**	**DR Induction**	**DR Control**	**fully fed Induction**	**fully fed Control**
dGCN2 overexpression	422	354	388	400
dGCN2 knockdown	391	483	482	490
dATF4 overexpression	441	376	487	487
dATF4 knockdown	466	492	366	363
B)		**Induction vs Control Statistics**		
**Condition**	**estimate**	**exp(estimate)**	**se**	**p-value**
dGCN2 overexpression	5.590	267.752	0.281	< 0.0001
dGCN2 knockdown	-0.238	0.788	0.128	0.0628
dATF4 overexpression	1.186	3.273	0.169	< 0.0001
dATF4 knockdown	-0.549	0.577	0.120	< 0.0001
C)		**DR vs fully fed Statistics**		
dGCN2 overexpression	-1.647	0.193	0.195	< 0.0001
dGCN2 knockdown	-2.328	0.0975	0.138	< 0.0001
dATF4 overexpression	-3.434	0.0323	0.169	< 0.0001
dATF4 knockdown	-2.293	0.101	0.123	< 0.0001
D)		**Interaction Statistics**		
dGCN2 overexpression	0.754	2.124	0.258	< 0.01
dGCN2 knockdown	0.311	1.365	0.187	0.0958
dATF4 overexpression	-0.00127	0.999	0.215	0.995
dATF4 knockdown	0.07	1.082	0.161	0.624

Table S2. Sample sizes and statistics for dose response of *dATF4* and *dGCN2* overexpression.
(A) All sample sizes per treatment in *dATF4* overexpression and *dGCN2* overexpression. (B,C) Statistics for effect low and medium levels of conditional overexpression of *dATF4* (B) and *dGCN*2 (C) compared to high, as well as low vs controls. (B,C) Statistics calculated using *coxme* R package.

A) **Sample Sizes:**

**Condition**	**Control**	**50µM RU (low)**	**100µM RU (medium)**	**200µM RU (high)**
dATF4 overexpression	644	636	637	638
dGCN2 overexpression	435	477	468	450

B) ***dATF4* overexpression**

	**estimate**	**exp(estimate)**	**se**	**p-value**
low vs high	-0.961	0.383	0.134	< 0.0001
medium vs high	-0.250	0.779	0.131	0.0772
low vs control	0.716	2.047	0.131	< 0.0001

C) ***dGCN2* overexpression**

	**estimate**	**exp(estimate)**	**se**	**p-value**
low vs high	-0.682	0.506	0.122	< 0.0001
medium vs high	-0.213	0.808	0.121	0.0559
low vs control	4.131	62.229	0.231	< 0.0001

Table S3. Sample sizes and statistics for fat body specific overexpression and knockdown of *dATF4* on DR. A) All sample sizes per treatment for *S106GS* (fat body-specific) *dATF4* overexpression and *dATF4* knockdown. B) Statistics for the effect of fat body specific *dATF4* overexpression and *dATF4* knockdown. Statistics calculated using the *coxme* R package.

A) **Sample Sizes:**

**Condition**	**DR Control**	**DR Induction**
dATF4 overexpression	314	298
dATF4 knockdown	380	477

B) **Induction vs Control Statistics**

	**estimate**	**exp(estimate)**	**se**	**p-value**
dATF4 overexpression	0.0929	1.097	0.0856	0.278
dATF4 knockdown	-0.233	0.792	0.0985	0.018

Table S4. Sample sizes and statistics for the effect of borrelidin treatment on *dATF4* manipulation. A, B) All sample sizes per treatment in (A) *dATF4* overexpression and (B) dATF4 knockdown using daGS driver. C-D) Model following selection using backward removal of non-significant terms. (C) dATF4 overexpression and (D) dATF4 knockdown. B-D Statistics calculated using the *coxme* R package. Note, sample sizes for fully fed (8% yeast) conditions are relatively low, and estimates are less certain for those treatments.

A) **Sample Sizes: *dATF4* overexpression**

**Condition**	**DR Induction**	**DR Control**	**Fully fed Induction**	**Fully fed Control**
Control	371	327	377	322
Borrelidin	304	386	249	271

B) **Sample Sizes: *dATF4* knockdown**

**Condition**	**DR Induction**	**DR Control**	**Fully fed Induction**	**Fully fed Control**
Control	336	326	161	147
Borrelidin	236	262	165	166

C) ***dATF4* overexpression**

	**estimate**	**exp(estimate)**	**se**	**p-value**
Borrelidin	0.329	1.39	0.148	0.038

Induction	1.91	6.75	0.220	<0.0001
DR	-1.35	0.260	0.238	<0.0001
Borrelidin:Induction	-0.950	0.387	0.232	<0.0001
Borrelidin:DR	0.139	1.15	0.289	0.64
Induction:DR	-1.02	0.361	0.327	0.002
Borrelidin:Induction:DR	0.843	2.32	0.417	0.04
**DR only**				
Borrelidin	0.470	1.60	0.259	0.07
Induction	0.852	2.34	0.270	0.002
Borrelidin:Induction	-0.249	0.780	0.383	0.52
**Fully fed only**				
Borrelidin	0.349	1.417	0.170	0.04
Induction	2.31	10.1	0.290	<0.0001
Borrelidin:Induction	-1.25	0.288	0.251	<0.0001

D) ***dATF4* knockdown**

	**estimate**	**exp(estimate)**	**se**	**p-value**
Borrelidin	-0.093	0.911	0.145	0.52
DR	-1.32	0.266	0.142	<0.0001
Induction	-0.214	0.807	0.079	<0.01
Borrelidin:DR	0.584	1.794	0.181	0.001
**DR only**				
Borrelidin	0.348	1.42	0.099	<0.001
Induction	-0.319	0.727	0.087	<0.001
Borrelidin:Induction	0.265	1.30	0.131	0.04

**Fully fed only**				
Borrelidin	0.067	1.07	0.308	0.83
Induction	-0.059	0.943	0.277	0.83
Borrelidin:Induction	-0.391	0.676	0.391	0.32

Table S5. Sample sizes and statistics for effect of Tyr on dATF4 knockdown on DR. A) All sample sizes per treatment group of *dATF4* knockdown. B) Statistics for the effect of Tyr supplementation in *dATF4* knockdown on DR (2% yeast) using *daGS* driver. Statistics calculated using the *coxme* R package.

A) **Sample Sizes:**

**Condition**	**Control**	**Induction**	**Control + Tyr**	**Induction + Tyr**
dATF4 knockdown	669	626	685	652

B) **Statistics**

	**estimate**	**exp(estimate)**	**se**	**p-value**
Induction	-0.439	0.645	0.103	< 0.0001
Tyrosine	-0.245	0.783	0.102	0.016
Interaction	0.295	1.343	0.145	0.041
Control + Tyr vs Control	-0.245	0.783	0.102	0.016
Induction + Tyr vs Induction	0.050	1.051	0.103	0.626

Table S6. Sample sizes and statistics for the effect of antibiotic treatment on *dATF4* manipulation.
A, B) All sample sizes per treatment in (A) *dATF4* overexpression and (B) *dATF4* knockdown using *daGS* driver. C-D) Statistics for the effect of antibiotics in (C) *dATF4* overexpression and (D) *dATF4* knockdown. B-D Statistics calculated using the *coxme* R package.

A) **Sample Sizes: *dATF4* overexpression**

**Condition**	**DR Induction**	**DR Control**	**Fully fed Induction**	**Fully fed Control**
Control	322	434	429	427
Antibiotics	458	478	340	448

B) **Sample Sizes: *dATF4* knockdown**

**Condition**	**DR Induction**	**DR Control**	**Fully fed Induction**	**Fully fed Control**
Control	347	356	358	349
Antibiotics	241	360	443	484

C) ***dATF4* overexpression**

	**estimate**	**exp(estimate)**	**se**	**p-value**
Antibiotics	0.129	1.138	0.132	0.330
RU	1.128	3.091	0.104	< 0.0001
Antibiotics:RU	-0.074	0.928	0.194	0.702

D) ***dATF4* knockdown**

	**estimate**	**exp(estimate)**	**se**	**p-value**
Antibiotics	0.012	1.012	0.106	0.913
RU	-0.364	0.695	0.109	< 0.001
Antibiotics:RU	0.023	1.023	0.1522	0.880

Table S7. Sample sizes and statistics for dATF4 overexpression and knockdown replicates.
A, B) All sample sizes per replicate in (A) *dATF4* overexpression and (B) *dATF4* knockdown using daGS driver. C,D) We analysed using coxme the effect of the induction of the transgene for each replicate, whilst controlling for diet. The main effect and the interaction with diet are reported. (C) *dATF4* overexpression and (D) *dATF4* knockdown.

A)

	Sample Sizes: *dATF4* overexpression			
**Condition**	**DR Induction**	**DR Control**	**8% Induction**	**8% Control**
Main	441	376	487	487
Antibiotics, control	322	434	429	427
Borrelidin control	371	367	377	322

B)

	Sample Sizes: *dATF4* knockdown			
**Condition**	**DR Induction**	**DR Control**	**8% Induction**	**8% Control**
Control	466	492	366	363
Antibiotics, control	347	356	358	349
Borrelidin, control	336	326	161	147

C) ***dATF4* overexpression**

	**estimate**	**exp(estimate)**	**se**	**p-value**
Main, Induction	1.186	3.273	0.169	< 0.0001
Main, Induction:DR	-0.00127	0.999	0.215	0.995
Antibiotics, control, Induction	1.101	3.008	0.186	< 0.0001
Antibiotics, control, Induction:DR	0.095	1.100	0.272	0.727
Borrelidin, control, Induction	1.862	6.441	0.205	< 0.0001
Borrelidin, control, Induction:DR	-0.844	0.420	0.285	< 0.01

D) ***dATF4* knockdown**

	**estimate**	**exp(estimate)**	**se**	**p-value**
Main, Induction	-0.549	0.577	0.120	< 0.0001
Main, Induction:DR	0.07	1.082	0.161	0.624
Antibiotics, control, Induction	-0.203	0.816	0.104	< 0.05
Antibiotics, control, Induction:DR	-0.355	0.701	0.140	< 0.05
Borrelidin, control, Induction	-0.247	0.781	0.119	< 0.05
Borrelidin, control, Induction:DR	-0.300	0.741	0.242	0.215

Table S8. Genes that changed significantly ($p < 0.01$) in expression and in opposite directions across both conditions (*dATF4* overexpression and knockdown). Annotation and the nearest human paralog was collated from Flybase. When prior literature appeared to link the gene to ATF4, this reference is listed and a short conclusion is provided. Literature search was conducted with the gene name and ATF4 and a quick manual scan of the associated literature in both Google and Google Scholar.

	***dATF4* overexpression**				***dATF4* knockdown**						
**Fly Base ID**	**logFC**	**PValue**	**FDR**	**symbol**	**logFC**	**PValue**	**FDR**	**annotation**	**human paralog**	**reference**	**conclusion**
FBgn01976	-0.47	0.001	0.023	Psf2	0.37	0.009	0.138	initiation of DNA replication	GINS2		
FBgn0261524	-0.37	0.009	0.089	lic	0.41	0.005	0.110	MAP kinase kinase	MAP2K3		
FBgn0022224	-0.28	0.009	0.089	ubl	0.34	0.002	0.079	Ubiquitin-like	UBL5	1	thought to be independent death pathway of ATF4-CHOP
FBgn0035805	0.25	0.010	0.094	CG7506	-0.33	0.002	0.084	participates in the c-ring assembly of mitochondrial ATP synthase	TMEM70		
FBgn0005278	0.31	0.010	0.094	Sam-S	-0.33	0.010	0.142	S-adenosylmethionine synthetase	MAT2A	2	appears known ATF4 target
FBgn0029755	0.32	0.004	0.060	Sas10	-0.41	0.001	0.078	Ribosomal biogenesis	UTP3		No direct connection but ribosomal biogenesis fits longevity effect
FBgn0015221	0.34	0.009	0.089	Fer2LCH	-0.37	0.005	0.111	Iron storage, one of the two subunits of insect ferritin		3	ATF4 regulates ferroptosis
FBgn0028473	0.48	0.002	0.042	Non1	-0.44	0.006	0.119	GTP binding protein	GTPBP4		
FBgn0032144	0.55	0.007	0.078	CG17633	-0.93	< 0.0001	0.005	Proteolysis, metallocarboxypeptidase activity	CPA1		
FBgn0035666	0.63	0.007	0.078	Jon65Aii	-0.64	0.007	0.127	Proteolysis and immune system			
FBgn0034871	0.70	0.002	0.040	CG3906	-0.73	0.002	0.079	unknown			
FBgn0036007	0.99	0.000	< 0.0001	path	-0.38	0.009	0.137	amino-acid transporter	SLC36A1	4	ATF4 regulates amino acid transporters and this likely include SLC36A1
FBgn0033446	1.25	0.000	0.008	CG1648	-1.01	0.002	0.081	unknown			

Supplementary References

1. Wang, W. et al. (2023) Ubiquitin-like protein 5 is a novel player in the UPR–PERK arm and ER stress–induced cell death. The Journal of biological chemistry, 299(7). https://doi.org/10.1016/j.jbc.2023.104915.
2. Chen, L. et al., (2021).Activating transcription factor 4 regulates angiogenesis under lipid overload via methionine adenosyltransferase 2A-mediated endothelial epigenetic alteration. The FASEB journal, 35(6), pp. e21612-n/a. https://doi.org/10.1096/fj.202100233R.
3. Tang, H. et al. (2024) ATF4 in cellular stress, ferroptosis, and cancer. Archives of toxicology, 98(4), pp. 1025–1041. https://doi.org/10.1007/s00204-024-03681-x.
4. Zhang, N. et al. (2018). Autophagy-deficient tumor cells rely on extracellular amino acids to survive upon glutamine deprivation. Autophagy, 14(9), pp. 1652–1653. https://doi.org/10.1080/15548627.2018.1493314.
